# Hemangioblastoma in the lateral ventricle: An extremely rare case report

**DOI:** 10.3389/fonc.2022.948903

**Published:** 2022-08-10

**Authors:** Ruihan Pan, Jianwei Shi, Yang Xu, Fuduo Bo, Zhengxiang Luo, Yansong Zhang

**Affiliations:** ^1^ First Affiliated Hospital, Zhejiang Chinese Medical University, Hangzhou, China; ^2^ Nanjing Brain Hospital Affiliated to Nanjing Medical University, Nanjing, China

**Keywords:** hemangioblastoma, lateral ventricle, magnetic resonance imaging, case report, neurosurgery

## Abstract

Hemangioblastoma (HB) is a benign vascular tumor that accounts for approximately 2% of intracranial neoplasms. HB of the lateral ventricles is extremely rare. Only a few reports are present in the literature. This article reports a 27-year-old male patient who arrived at our hospital because of a progressive headache lasting one month. The brain Magnetic Resonance Imaging (MRI) revealed a solid-cystic mass of 3.5×3.0 cm in size located in the left lateral ventricle, the surgery was performed by applying an interhemispheric approach to single frontal craniotomy with coronal incision to remove the tumor. The postoperative CT and MRI showed the successful complete removal of the tumor and a normal ventricle morphology. The differential diagnosis should be considered in case of intraventricular tumors including HB. Angiography should be performed prior to surgery when HB is suspected.

## Introduction

Hemangioblastoma (HB) is a benign tumor that usually occurs in the posterior fossa of the central nervous system (CNS) and is more common in adults. This tumor is associated with von Hippel-Lindau disease (VHL) which is an autosomal dominantly inherited neoplastic disorder that demonstrates marked phenotypic variability and age-dependent penetrance. CNS HB occur in 60–80% of VHL patients and the remaining are sporadic cases (1). HB is responsible for approximately 2% of the intracranial tumors and 8% to 12% of the posterior fossa tumors (2, 3). Supratentorial HB is rare and only a few reports are available on the intraventricular location of a tumor, especially in the lateral ventricles (4, 5). In addition, only a few reported cases of solitary intraventricular HB of the lateral ventricles are available. Therefore, this work describes a case of HB located in the lateral ventricle based on the obtained imaging features and confirmed by pathology.

## Case presentation

**Figure 1:**
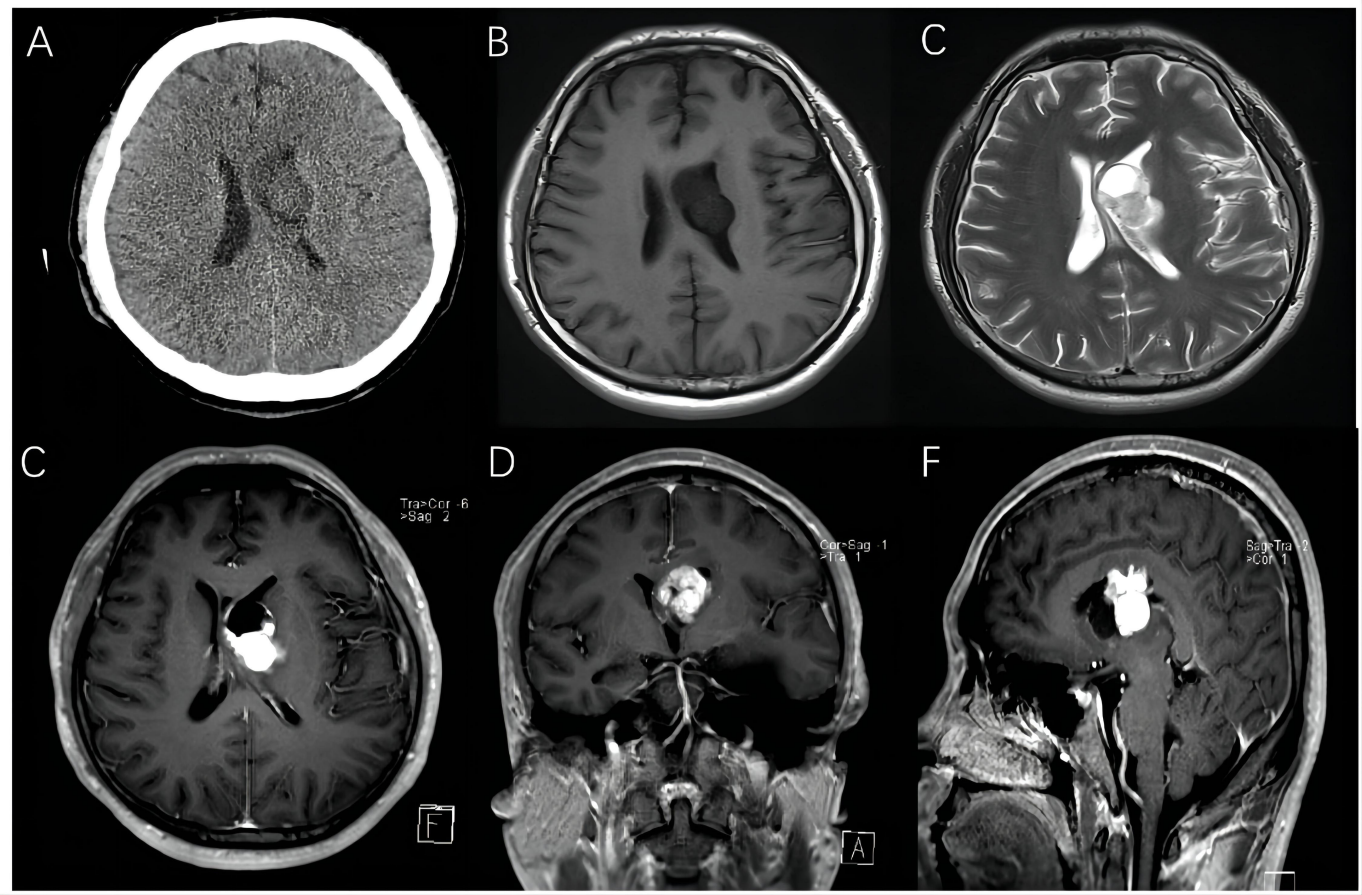
Preoperative images of a 27-year-old man with intraventricular hemangioblastoma. **(A)** CT scans showed a 3.5 cm hypo-isodense mass located in the left lateral ventricle. **(B–F)** MRI revealed a solid-cystic mass, and the solid part of mass was mildly heterogeneously enhancing.

A 27-year-old male patient arrived at our hospital with a 1-month history of progressive headache. No neurologic deficits were found, and his medical history was not significant according to the physical examination. The brain computed tomography (CT) revealed the presence of a 3.5×3.0 cm hypo-isodense mass located in the left lateral ventricle. The magnetic resonance imaging (MRI) revealed a large solid-cystic mass extended from the left internal capsule to the left lateral ventricle. The ventricular septum was squeezed to the right. The mass was hypo-isointense on T1-weighted images and hyper-intense on T2-weighted images. The solid part of the mass was slightly heterogeneously enhanced after enhancement (**Figure 1**).

**Figure 2:**
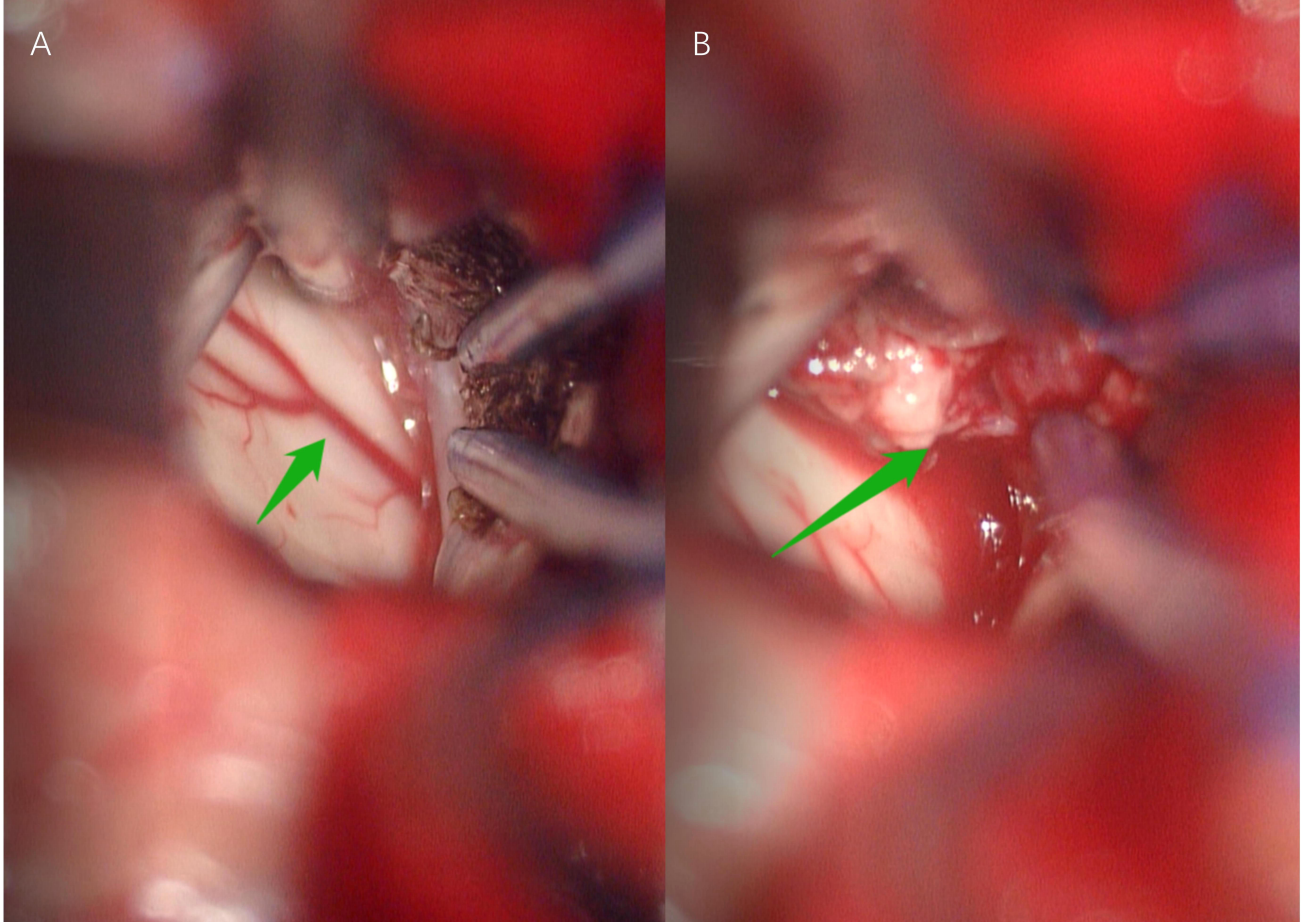
Anterior interhemispheric approach for hemangioblastoma. Arrow in **(A)** Arteria pericallosal. Arrow in **(B)** Intraventricular tumor with abundant blood supply, unclear boundary and brittle texture .

**Figure 3:**
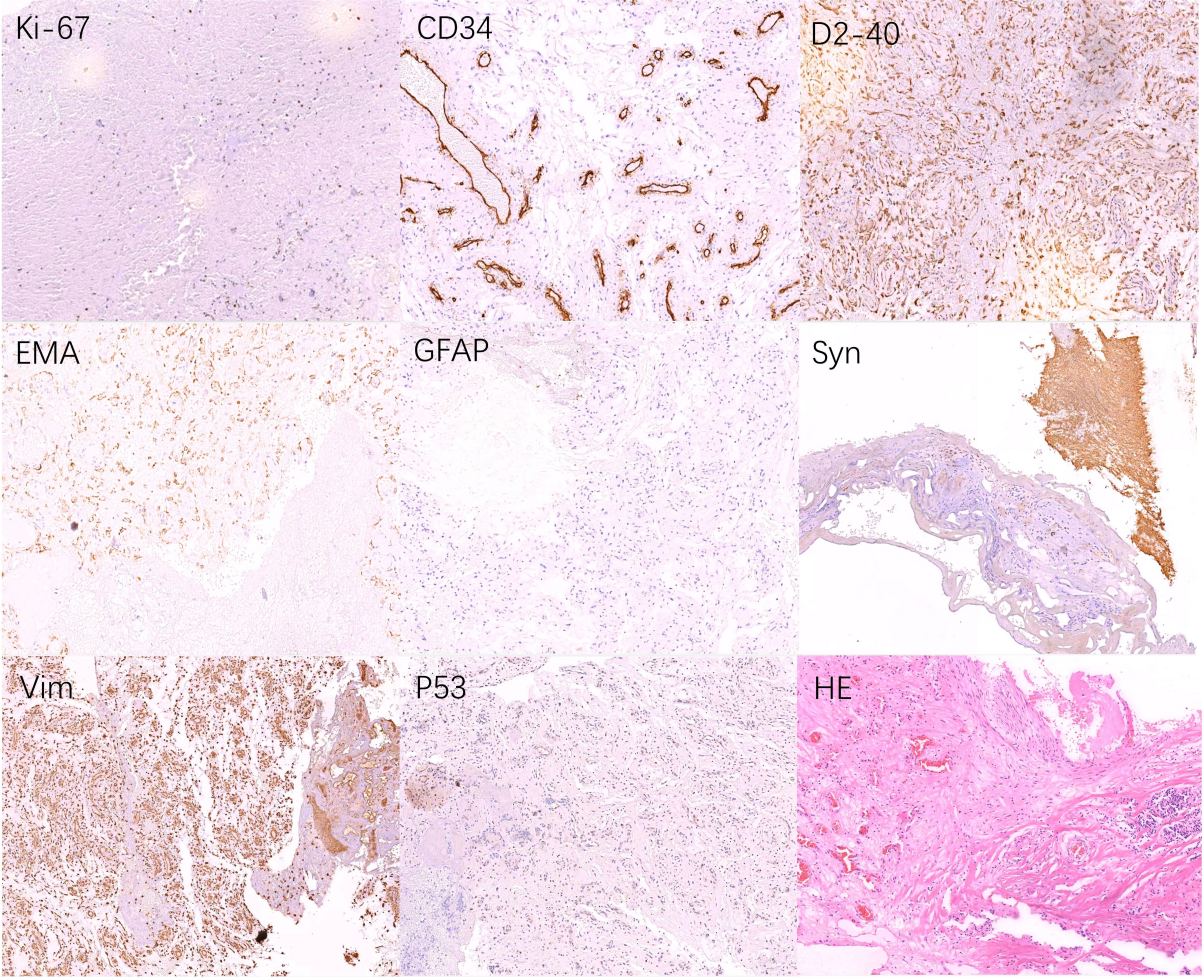
Pathological examination after surgery. Tumors were composed of capillaries and stromal cells of different maturation stages, some of which are mildly atypical, we considered hemangioblastoma (WHO grade I) as the final diagnosis. Tumor tissue: Ki-67(2%+), P53(<1%+), CD56(-), NSE(-), Syn(+), EMA(+), CgA(-), PR(-), SALL4(-), GFAP(+\-), S-100(--), CKpan(-), Vim(+), Myoglobin(-), CD34(Endothelial cells+), D2-40(+), TTF-1(-), Inh-α(-), CD10(-).

Our primary preoperative differential diagnosis according to the MRI features was an intraventricular subependymoma. Other potential diagnoses were glioma and central neurocytoma, while HB was not included in the differential diagnosis before surgery at the time of the initial diagnosis. The surgery was performed by realizing an interhemispheric approach to single frontal craniotomy with coronal incision to remove the tumor. When the tumor bulk was reached during surgery, a large mass was found, which was highly vascular and hemorrhagic (**Figure 2**). Finally, a ventricular drain was placed into the left lateral ventricle after removing all the visible parts of the tumor and after performing a meticulous intraventricular hemostasis. The postoperative brain CT scan showed the total removal of the tumor. The histopathological results of the mass indicated HB, which was not considered among our preoperative diagnoses (**Figure 3**).

**Figure 4:**
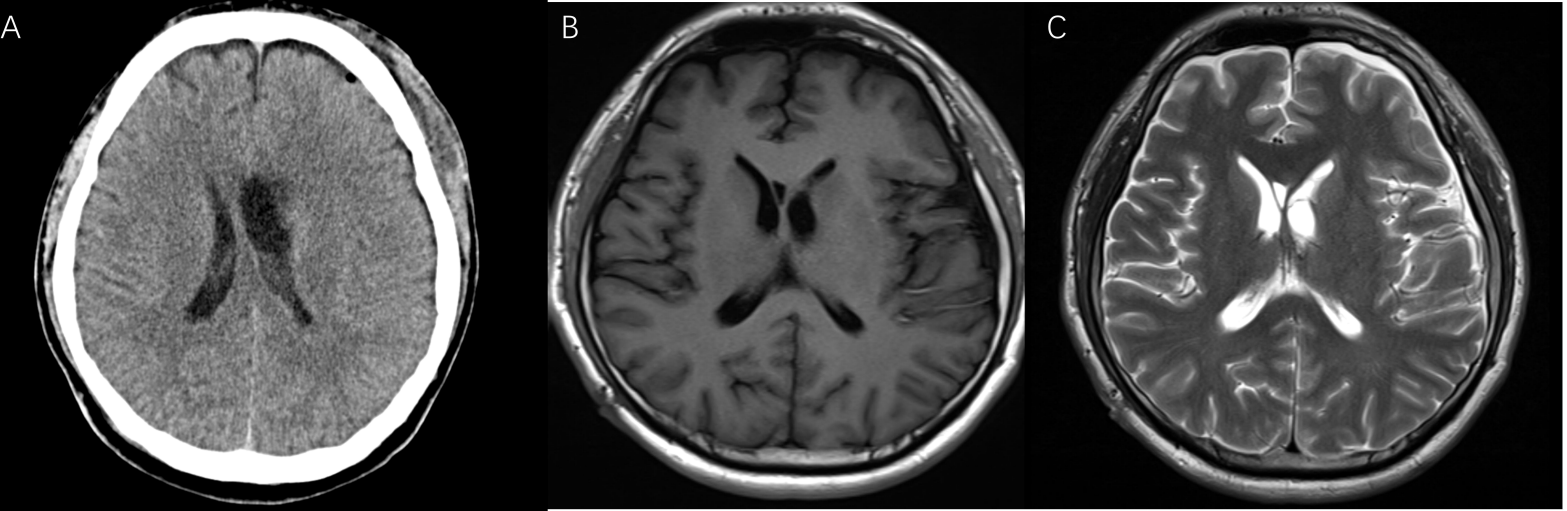
The postoperative CT **(A)**, T1-weighted **(B)**, and T2-weighted MRI **(C)** after one month showed good tumor resection.

After the surgery, the patient was in good health without neurological deficits and did not show any of the signs and symptoms previously reported. The follow-up after one month still revealed the absence of the tumor with normal ventricle morphology as shown by CT and MRI (**Figure 4**).

## Discussion

Lateral ventricle tumors represent a small part of intracranial tumors, but they include many different biological types. The vast majority of the lateral ventricle tumors are benign with slow progression and are usually accidentally found because caused by symptoms of compression and hydrocephalus (6). However, the origin of the tumor and the age of the patients are somehow indicating a specific type of tumor according to published articles and shared experiences. Children are likely to develop choroid plexus papilloma, which occurs in the body and in the triangle area of the lateral ventricle. Hydrocephalus may be caused by this type of tumor, due to the excessive secretion of cerebrospinal fluid by the excessive stimulation of the choroid plexus (7). Meningiomas are usually found in the triangle area of the lateral ventricle in adults, with tumor calcification instead of cystic changes (8). Besides, ependymoma are CNS tumors and usually occurs in children (9). The treatment and prognosis of tumors vary greatly in line with their pathological nature. Therefore, a correct classification and evaluation of the lateral ventricle tumors are of utmost importance in guiding the surgical plan and long-term prognosis.

However, reports on HB in the lateral ventricle remain insufficient (10, 11). HB is a rare intracranial benign tumor originating from the remaining embryonic tissue composed of mesoderm cells, and it is a true vascular tumor. Microscopically, haemangioblastomas consist of large polygonal stromal cells enmeshed in a capillary network and stromal cells arise from mesoderm-derived embryologically arrested haemangioblasts (12). In 2016, WHO classified it as other types of tumors related to meninges (13). The incidence of HB is second only to medulloblastoma and astrocytoma, accounting for 1.1%-2.4% of the CNS tumors and 7%-12% of the posterior fossa tumors. This tumor is more common in young and middle-aged men, and the incidence rate in males and females is approximately 2:1 (14). It is mostly located in the infratentorial area, common in the cerebellum, but also in the brainstem and spinal cord. It is rarely observed in the supratentorial, and HB of the lateral ventricles is very rare (15). Only a few reports on lateral ventricular HB are available, as shown in **Table 1**. In our patient, HB was located in the left lateral ventricle, with imaging feature as cystic-solid type.

**Table 1:** Previous studies reporting HB cases.

Author	Sex, age(years)	Signsand symptoms	Site	Preoperative imaging features	Diameter (cm)
Vecchi et al. (1935) (16)	Female, 80	Incidental postmortem	N/A	N/A	N/A
Diehl et al. (1981) (17)	Male, 20	Progressive visual loss, headache, bilateralpapilledema	Right temporal horn	CT scan,homogenous	5
Murakami et al. (1985) (18)	Female, 31	Headache, gait disturbance, bilateralpapilledema	Trigone of rightlateral ventricle	CT scan,homogenous	N/A
Ho et al. (1990) (11)	Female, 44	Headache	Trigone of rightlateral ventricle	MRI,homogenous	3
Lyo et al. (1992) (19)	Male, 36	Headache, diplopia, right abducens nerve palsy,confusion	Left lateral ventriclewall	N/A	N/A
Prieto et al. (2005) (10)	Male, 37	Confusion, cognitive deficit, dysphasia	Trigone of left lateral ventricle	MRI, homogenous	3
Jaggi et al. (2009) (20)	Male, 30	Headache	Left lateral ventricle	MRI,homogenous	3
Takeuchi et al. (2011) (21)	Male, 33	Headache	Right lateralventricle	MRI, solid-cystichomogenous	N/A
Al-Najar et al. (2013) (22)	Male, 70	Headache, vomiting, dizziness, left facial palsy, left hemiparesis, intraventricularhemorrhage	Right occipital horn	MRI,heterogeneous	3.3
Anderson et al. (2014) (23)	Male, 24	Vertigo, right arm weakness, transient right facial paresis and slurringof speech, headache	Midline and greater in left lateralventricle	MRI, solid-cystic with diffuseenhancement	N/A
Alireza Tabibkhooei et al. (2020) (24)	Male, 30	Seizure, blurred vision, headache, bilateralpapilledema	Midline in bothlateral ventricle	MRI,heterogeneous	5.5
**Present case (2022)**	**Male, 27**	**headache**	**Left lateral ventricle**	**MRI, solid-cystic with diffuse enhancement**	**3.5**

N/A, not available.

The imaging manifestations of supratentorial and infratentorial HB are basically the same, and they are generally divided into three types: cystic-solid type, simple cystic type, and solid mass type. The supratentorial cystic-solid HB is mainly differentiated from low-grade astrocytoma, which shows mild enhancement and no peritumoral vascular shadows. However, cystic-solid HB generally has an evident enhancement, with vascular shadows around the tumor. It is also different from simple intracerebral cysts and arachnoid cysts. The signal of a simple cyst is similar to that of cerebrospinal fluid in each MRI sequence, with no vascular flow shadow around the cyst, while each sequence image of HB is higher than the cerebrospinal fluid signal shadow, indicating a potential vascular flow shadow around the cyst.

HB is closely related to Von Hippel-Lindau (VHL) disease. VHL is an autosomal dominant genetic disorder with 3 common types. Type I includes retinal and central nervous system HB (CNS-HB), renal cysts, renal carcinoma, and pancreatic cysts. Type II includes pheochromocytoma and pancreatic tumor of the islet cells, besides retinal HB and CNS-HB. Type III is less common and includes retinal HB, CNS-HB and pheochromocytoma (25). Generally, patients suffering from VHL are young and they genetically inherited it. Intracranial lesions can be multiple, accompanied by other diseases including retinal hemangioma and renal cysts. Therefore, children and adolescents should be screened for systemic lesions or VHL (26). In this patient, the possibility of VHL was excluded by abdominal B-ultrasound and fundus examination.

Surgery is currently the preferential option in the treatment of HB, although radiation and medication therapy are also considered. Preoperative embolization is of great use to reduce the risk of intraoperative bleeding due to the extremely rich blood supply, especially for solid tumors (27). Stereotactic radiotherapy provides good control of tumor growth and neurological protection but is not suitable for multiple lesions. Besides, inhibitors of vascular endothelial growth factors that block tumor angiogenesis may be considered as a novel treatment option (28).

Taken together, our report presented a rare case of HB in the lateral ventricle. The most relevant differential diagnosis of a mass in the lateral ventricle in this particular age group includes subependymoma, which is commonly associated with hydrocephalus and often presenting cystic components and calcifications but rarely presenting hemorrhage. Therefore, according to these characteristics (29), the disease in this patient was misdiagnosed as subependymoma before surgery. Angiography was not performed and consequently, our patient had severe intraoperative bleeding. Notably, VHL genetic screening is recommended for young patients and their family members. However, more cases should be described and research is in urgent need to provide a diagnostic basis and systematic treatment experience.

## Conclusion

A patient with HB in the left lateral ventricles was described in this report. The MRI showed a regularly enhanced imaging in the solid part of the tumor. The main disadvantage of this case was the excessive intraoperative bleeding. Therefore, a preoperative angiography is recommended for the detection of large intraventricular cystic-solid masses. The differential diagnosis should be considered in case of intraventricular tumors including HB.

## Data availability statement

The original contributions presented in the study are included in the article/supplementary material. Further inquiries can be directed to the corresponding author.

## Ethics statement

Written informed consent was obtained from the individual(s) for the publication of any potentially identifiable images or data included in this article.

## Author contributions

RP, JS, and YZ performed the literature search; RP, JS, YX, and FB prepared the manuscript for submission; ZL contributed to the section on radiology; YZ was the lead clinician/surgeon and performed the operations described. All authors reviewed the manuscript. All authors contributed to the article and approved the submitted version.

## Conflict of interest

The authors declare that the research was conducted in the absence of any commercial or financial relationships that could be construed as a potential conflict of interest.

## Publisher’s note

All claims expressed in this article are solely those of the authors and do not necessarily represent those of their affiliated organizations, or those of the publisher, the editors and the reviewers. Any product that may be evaluated in this article, or claim that may be made by its manufacturer, is not guaranteed or endorsed by the publisher.
